# Revisiting the Cerebellum's Linguistic Role: Evidence for Cerebellar Involvement in Expressive Syntax

**DOI:** 10.1007/s12311-025-01879-y

**Published:** 2025-07-10

**Authors:** Melanie Esver, Caitlin Cloud, Allison Hilger, Christine Brennan

**Affiliations:** 1 University of Colorado Boulder, 2501 Kittredge Loop Drive, Boulder, CO 80039 USA; 2 Present Address: Therapy Place 4 Kids, 1932 14th St, Santa Monica, CA 90404 USA; 3 Present Address: University of Nebraska-Lincoln, 301 Barkley Memorial Center, Lincoln, NE 68583-0738 USA

**Keywords:** Cerebellar physiology, Dysarthria, Syntax, Speech-language pathology

## Abstract

The cerebellum’s role in language remains unclear, though cerebellar damage may influence expressive language via neurological or compensatory mechanisms. This study investigated the syntactic complexity of spoken language in individuals with dysarthria due to cerebellar ataxia. Speech samples were collected via a picture description task from 26 individuals with cerebellar ataxia and 28 age- and sex-matched controls. Two complete sentences from each participant’s picture description were selected for analysis and categorized by the presence of dependent clauses. A novel syntactic complexity scoring system, based on the developmental acquisition of parts of speech and validated against *Systematic Analysis of Language Transcripts* (SALT) scoring, was used to analyze a total of 108 utterances. The novel complexity scores showed high correlation with SALT scores, supporting validity. While overall syntactic complexity was higher in control participants (Cohen’s d = 0.39), the difference was not statistically significant (p = 0.057). However, when analyzed by clause type, controls produced significantly more complex utterances for both independent and dependent clauses. Individuals with ataxia also produced fewer dependent clauses overall. Within the ataxia group, syntactic complexity was not associated with dysarthria severity, naturalness ratings, dysarthria impact, age, or sex. This study suggests a potential contribution of cerebellar dysfunction to reduced expressive syntactic complexity, based on significant group differences observed in specific clause types. Although the overall group difference in syntactic complexity did not reach statistical significance, the results highlight patterns consistent with a cerebellar role in language formulation. This work also introduces and validates a new tool for quantifying syntactic complexity in connected speech, which may support future research in disordered language production.

**Supplementary Information:**

The online version contains supplementary material available at 10.1007/s12311-025-01879-y.

## Introduction

Language is the process of transforming often nonsequential thoughts into words and sounds, sign gestures, or orthographic forms. Syntax, a fundamental component of language, encodes hierarchical relationships between words, enabling infinite meaning to be conveyed through finite constructions [39]. An important goal of linguistic research has been to determine neural processing regions for syntax. While traditional models have focused on cortical structures such as Broca’s area and Wernicke’s area as primary language centers [18, 21, 39], recent research suggests that subcortical structures, particularly the cerebellum, may play a role in linguistic processing beyond motor execution [6, 22, 28, 37], 2014).

This shift in perspective is particularly relevant in the study of ataxic dysarthria (AD), a motor speech disorder resulting from cerebellar impairment. Dysarthria encompasses a family of speech disorders resulting from neurological injury that impairs the execution of speech [69]. AD, specifically, results from cerebellar impairment, which affects the timing, scaling, and coordination of movement [29, 30]. The speech abnormalities seen in AD, such as inconsistent loudness, variable stress patterning, and vowel distortion, closely mirror the known coordination functions of the cerebellum [61]. While intelligibility is often minimally impacted [23], individuals with AD frequently experience disrupted speech naturalness and efficiency, significantly impacting their quality of life [23, 24]. Although the motor speech deficits associated with AD are well-documented, the potential non-motor linguistic consequences of cerebellar damage are only beginning to be explored [6, 22, 37, 62]. Just as motor impairments affect communication in AD, non-motor impairments may also contribute to communicative difficulties, yet these linguistic effects remain poorly understood.

This study investigates the potential origins of reduced syntactic complexity in individuals with AD, with particular attention to whether such reductions reflect a core linguistic impairment due to cerebellar dysfunction or an adaptive response to speech-motor demands. To explore this, we address three central research questions: (1) Is there a measurable difference in the syntactic complexity of expressive language between individuals with AD and neurologically typical control speakers? (2) If syntactic simplification is observed, can it be attributed to underlying cerebellar pathology, or might it reflect compensatory strategies such as reduced sentence elaboration to ease articulatory effort? (3) Do individual differences in syntactic complexity correlate with demographic or clinical variables, including age, sex, dysarthria severity, speech naturalness, or dysarthria impact of the cerebellar disorder? Clarifying these relationships will contribute to a more nuanced understanding of the cerebellum’s role in language and may help refine intervention strategies for individuals with AD.

## Traditional Cortical Models vs. Emerging Cerebellar Involvement

For decades, Broca’s area, located in the posterior inferior frontal gyrus, has been considered a critical hub for syntactic processing. Damage to this area often results in expressive agrammatism, where individuals omit grammatical elements and simplify sentence structure [19, 20]. More recent research has identified the posterior temporal lobe as another crucial region for syntactic processing, challenging the notion that syntax is exclusively frontally localized [2, 12, 46, 47, 53, 67]. Additionally, the anterior temporal lobe has been implicated in syntactic and semantic functions [8]. White matter tracts connecting these regions are further posited to contribute to syntactic processing, although further investigation is needed to determine precisely how these cortical networks function together [59].

In contrast to these purely cortical models, recent studies suggest the cerebellum plays a role in broader cognitive and linguistic domains, including attentional control [14], working memory [38], affective regulation [57], and even language production [1, 11, 17, 28]. This growing body of evidence suggests that the cerebellum may contribute to syntactic and morphosyntactic processing, possibly through its connections to cortical language regions.

Functional neuroimaging and lesion studies have identified the posterior lateral cerebellum as a key region implicated in linguistic processing [62]. More recently, Guell et al. [22] provided converging task-based and resting-state fMRI evidence for a “triple representation” of language in the cerebellum, showing three distinct zones (Crus I, Crus II, and lobule IX) bilaterally that are functionally connected to canonical cortical language areas. These regions form closed-loop circuits with classical language areas in the inferior frontal and posterior temporal lobes via the cortico-ponto-cerebellar and cerebello-thalamo-cortical pathways [22, 37, 63]. Through these circuits, the cerebellum is believed to contribute to internal modeling, predictive control, and the monitoring of rule-based operations such as syntactic structuring. While the cerebellum likely does not store vocabulary or grammatical rules per se, its role in sequencing, working memory, and hierarchical prediction makes it well-suited to support syntactic formulation and real-time error correction during speech [28, 37, 62].

Studies indicate that cerebellar damage can lead to deficits in various cognitive and affective functions, including non-motor linguistic impairments [6]. Specifically, Bodranghien et al. [6] reported that individuals with cerebellar damage exhibited deficits in both receptive and expressive syntax. These findings suggest that reduced syntactic complexity in individuals with AD may be an inherent consequence of cerebellar dysfunction rather than solely a compensatory adaptation.

Research has increasingly explored the cerebellum’s role in syntax, with evidence suggesting that cerebellar damage impacts grammatical morphology and syntactic structuring [28]. Studies on patients with cerebellar dysfunction have found that reductions in speech output led to a lower proportion of closed-class words (i.e., “function words” such as articles, prepositions, and conjunctions), potentially due to their reduced importance in conveying core meaning. Additionally, some patients with cerebellar damage show a diminished ability to process grammatical morphology [28], particularly subject-verb agreement, similar to individuals with Broca’s aphasia [68].

More recent studies provide additional evidence for the cerebellum’s role in both expressive and receptive syntax. Mariën et al. [37] found that individuals with cerebellar disease demonstrated impairments in grammatical processing beyond speech-motor control. Furthermore, Schmahmann [56] reported that individuals with cerebellar disease performed poorer than controls during the Test of Language Competence-Expanded in all aspects of the metalinguistic assessment. These individuals also demonstrated diminished event-related potential (ERP) peaks when presented with syntactic errors. Recent evidence from stroke studies further supports the cerebellum’s role in core linguistic processing. In a prospective study of 43 individuals with isolated cerebellar stroke, Satoer et al. [55] found impairments in word retrieval, sentence repetition, semantic processing, and syntactic completion, independent of lesion laterality or volume. These findings suggest that the cerebellum may contribute not only to motor coordination in speech but also to the structural organization of language at a cognitive level.

Notably, patients with cerebellar ataxia demonstrate variability in their syntactic impairments. While some perform near-flawlessly on sentence-processing tasks, others struggle with word order errors and subject-verb agreement [28]. This heterogeneity suggests that cerebellar contributions to language may be more nuanced than previously assumed. For instance, while many patients with cerebellar damage produce canonical word orders, they often reduce the use of grammatical morphology, particularly in tasks requiring article use [28]. Such findings highlight the cerebellum's potential role in fine-tuning syntactic structures rather than generating them outright.

## Cerebellar Contributions Beyond Syntax

Beyond syntax, the cerebellum has been implicated in broader linguistic and cognitive functions. Studies have linked the cerebellum to verbal fluency [58], overall nonmotor roles [63], and speech production [1, 7, 10]. Additionally, cerebellar networks are involved in temporal processing and production of rhythmic tasks [27, 44] and even music perception and production [15, 16]. These findings collectively support the notion that the cerebellum is not solely a motor-regulating structure but plays a broader role in cognitive-linguistic functions [32, 37, 49].

## Current Study

The cerebellum’s proposed non-motor contributions to language, combined with the known use of compensatory strategies in individuals with AD, raise key questions about the underlying source of syntactic simplification in this population. This study aims to explore whether there is reduced syntactic complexity in individuals with AD, and if there is, whether it reflects a linguistic impairment linked to cerebellar dysfunction or an adaptive strategy to accommodate motor speech challenges.

Specifically, the study addresses three research questions:

1. Is there a measurable difference in the syntactic complexity of expressive language between individuals with AD and neurologically healthy control speakers?
2. If syntactic simplification is present, does it appear to result from cerebellar dysfunction or from compensatory strategies (e.g., economy of words) used to reduce speech effort?
3. Do demographic or clinical factors (including age, sex, dysarthria severity, speech naturalness, or dysarthria impact) correlate with syntactic complexity?

To investigate these questions, we analyzed picture description samples from speakers with AD and healthy controls, examining sentence types (e.g., independent and dependent clauses) and applying a validated syntactic complexity scoring system. This approach aims to clarify the linguistic profile of AD and assess how structural language patterns relate to neurological and speech-motor variables.

To systematically analyze syntactic complexity, we developed a novel scoring system that assigns complexity scores to individual utterances. This framework is informed by the developmental trajectory of syntax and morphology acquisition in English-speaking children. Language development research has demonstrated that more complex syntactic structures emerge later in life, providing a natural metric for evaluating sentence complexity [45]. For example, early language acquisition prioritizes simple noun-based utterances, whereas later development incorporates more advanced grammatical features such as phrasal structures and subordinating conjunctions. Our scoring system reflects these developmental principles, allowing for a structured analysis of syntactic production in speakers with AD. With this approach, we set out to answer whether reduced syntactic complexity is consistently present in individuals with AD. We hypothesized that speakers with AD may modify their expressive language to compensate for motor speech impairments, resulting in syntactic simplification. Furthermore, we predicted that syntactic complexity would correlate with dysarthria severity, with more severe cases demonstrating greater reductions in complexity. If our findings support these hypotheses, they will indicate that expressive language adaptations occur in AD even in the absence of an overt linguistic impairment. Understanding these patterns has important implications for both theoretical models of cerebellar involvement in language and clinical approaches to supporting communication in individuals with AD. While this study focuses on syntactic complexity in individuals with cerebellar ataxia, we acknowledge that many forms of cerebellar disease, particularly hereditary ataxias, may involve extracerebellar regions as well, complicating efforts to attribute observed language patterns exclusively to cerebellar function.

## Methods

This current paper is part of a more extensive study on auditory feedback control in cerebellar ataxia [25]. For the current paper, a contextual speech task was chosen to control conversational focus for the syntax analysis. Tasks obtained in the larger study are being utilized for future research.

### Participants

#### Individuals with Ataxic Dysarthria

Twenty-six participants with cerebellar ataxia (8 males, 18 females) were recruited for a prior study. Ages ranged from 24–79 years (M = 54.3, SD = 15.1). Education ranged from 12–22 years (M = 15.3; SD = 2.5). All participants were native speakers of American English. Participants had normal or corrected to normal visual acuity. Ataxia diagnosis was confirmed through participant self-reports of neurology or genetic testing. Participants were recruited through local support groups, outpatient clinics of local medical/rehabilitation facilities, flyers in the monthly National Ataxia Foundation newsletter [42], social media, word of mouth, the Communication Research Registry at Northwestern University, and the CoRDS registry [54],Coordination of Rare Diseases at Sanford). Summary characteristics of speakers with ataxia are provided in Table 1.

**Table 1:** Participant characteristics. Participants are listed by group (AT = ataxia, CO = control), participant number, sex (M = male, F = female), education, ataxia diagnosis (SCA = spinocerebellar ataxia, AOA = ataxia with oculomotor apraxia, SCAR = spinocerebellar ataxia recessive autosomal, FA = Friedreich’s Ataxia), disease duration, and dysarthria severity

Participant Group	Participant Number	Sex	Age	Education (years)	Ataxia Diagnosis	Disease Duration	Dysarthria Severity
AT	1	M	67	14	SCA-Unknown	2.5	Mild
AT	2	M	47	14	SCA-Unknown	23	Mild-Moderate
AT	3	M	72	22	SCA6	3	Severe
AT	4	F	62	14	SCA6	1	Mild-Moderate
AT	5	F	42	16	SCA2	0.5	Mild-Moderate
AT	6	M	36	12	SCA7	0.5	Mild
AT	7	M	55	14	SCA1	22	Severe
AT	8	M	24	14	SCA2	3	Mild
AT	9	F	67	16	SCA6	20	Mild-Moderate
AT	10	F	41	18	SCA3	10	Mild-Moderate
AT	11	F	55	14	SCA3	0.5	Mild
AT	12	F	63	14	SCA6	3	Mild
AT	13	F	69	15	SCA-Unknown	10	Moderate
AT	14	F	70	16	SCA3	5	Mild
AT	15	M	64	12	SCA15	24	Mild
AT	16	F	65	14	SCA-Unknown	7	Mild-Moderate
AT	17	F	62	18	FA	14	Mild
AT	18	F	36	18	SCA5	13	Mild
AT	19	F	42	18	AOA2	23	Mild-Moderate
AT	20	F	60	18	SCAR8	21	Mild-Moderate
AT	21	M	55	16	FA	14	Mild-Moderate
AT	22	F	55	18	SCA-Unknown	2	Moderate
AT	23	F	79	12	SCA-Unknown	3	Mild
AT	24	M	31	12	FA	0.5	Mild-Moderate
AT	25	F	47	18	SCA-Unknown	25	Mild-Moderate
AT	26	F	28	12	FA	12	Mild-Moderate
AT = Ataxia, CO = Control, SCA = Spinocerebellar Ataxia, FA = Friedreich’s Ataxia, M = Male, F = Female							
CO	1	M	68	18			
CO	2	M	45	16			
CO	3	M	71	18			
CO	4	F	61	12			
CO	5	F	38	18			
CO	6	M	38	18			
CO	7	M	55	18			
CO	8	M	24	16			
CO	9	F	65	16			
CO	10	F	40	16			
CO	11	F	51	12			
CO	12	F	66	18			
CO	13	F	70	22			
CO	14	F	70	18			
CO	15	M	63	18			
CO	16	F	63	18			
CO	17	F	60	18			
CO	18	F	36	22			
CO	19	F	41	18			
CO	20	F	58	18			
CO	21	M	50	18			
CO	22	F	71	16			
CO	23	F	79	16			
CO	24	F	54	18			
CO	25	M	36	18			
CO	26	F	42	18			
CO	27	F	23	20			
CO	28	F	62	18			

Dysarthria type and severity were assessed using the Frenchay Dysarthria Assessment (FDA-2) [13], a standardized assessment sensitive to various severity and subtypes of dysarthria. The FDA-2 assesses the level of function for speech subsystems, including respiration, articulation, phonation, resonance, and intelligibility. Dysarthria severity was assessed by comparing the level of function across the speech subsystems. All participants completed the Dysarthria Impact Profile, a patient-reported outcome measure designed to measure the psychosocial impact of acquired dysarthria. All participants were screened for a cognitive impairment cut-off score using the Montreal Cognitive Assessment (MoCA)[41]. Only one participant received a score below the cut-off used for this study; all other participants scored within the normal range. This study was approved by the Northwestern University Institutional Review Board (IRB). All participants provided written informed consent prior to participating in the study. Research procedures involving human participants were conducted in accordance with the ethical standards of the Northwestern University IRB and with the 1964 Declaration of Helsinki and its later amendments or comparable ethical standards. This study was not a clinical trial (clinical trial number: not applicable).

#### Healthy Control Speakers

Twenty-eight adults with no reported history of speech, language, or neurological impairment were recruited for this study as age- and sex-matched control participants (10 males, 18 females). All participants were native speakers of American English. Ages ranged from 24–71 years (M = 54.1, SD = 15.0). Years of education ranged from 12–22 years (M = 17.3; SD = 2.1). Participants had normal or corrected to normal visual acuity. Participants passed hearing and cognitive screenings.

### Experiment Overview

#### Speech Sample

Spontaneous speech samples were elicited from participants for conversational speech (from the prompt, “Tell me about a typical day”), passage reading (from the Grandfather Passage; [51], and picture description (from the Cookie Theft Picture; [26]). For this study, only speech samples from the picture description task were analyzed, as it provides a structured yet flexible context that allows for variation in sentence production. Speakers were shown the picture and prompted with the following “*Tell me what is going on in this picture for 2–3 min.*” Participants spoke into an over-ear microphone (AKG, model C420) positioned approximately one inch from the corner of the mouth. Recordings of the microphone signal were obtained using a multi-channel recording system (AD Instruments, model ML785, PowerLab A/D converter) and LabChart software (AD Instruments, v.7.0) with a sampling rate of 20 kHz. Following these speech recordings, participants with ataxia completed the Dysarthria Impact Profile [66].

#### Syntactic Complexity Analysis

The picture description task was transcribed in ELAN (Version 6.3), which aligned the transcriptions with the audio file through manual segmentation by phrase. The resulting transcriptions were then exported as datasheets with specific time domains listed for each annotated phrase. For this analysis, two complete sentences from each participant’s picture description were randomly selected. A sentence was defined as meeting one of the following two criteria: (a) an independent clause with any associated dependent clauses, or (b) two independent clauses connected by a subordinating conjunction. This ensured that selections were full sentences rather than incomplete phrases, while also preventing artificially long sentences resulting from participants linking multiple independent clauses with coordinating conjunctions like “and.” To ensure unbiased sentence selection, a random number generator was used to generate integers corresponding to the line numbers of transcribed sentences. Each selected sentence was then reviewed to confirm that it met predefined criteria for a complete sentence (i.e., an independent clause with or without dependent clauses, or two independent clauses joined by a subordinating conjunction). If a selection did not meet these criteria, a new sentence was randomly chosen.

The speech phrases were broken into independent and dependent clauses. Dependent clauses were determined by identifying phrases following subordinating clauses or phrases that would stand as incomplete sentences in isolation. The selected sentences were run through a custom script in Python (version 3.9.2), which categorized all words within the sentences into parts of speech types using functions available in the Natural Language Toolkit (NLTK) package [5]. The accuracy of categorization was confirmed and adjusted as needed by two research assistants. A frequency of occurrence count was taken for each part of the speech category within the groups. These data can be seen in Supplemental Table 1.

A syntactic complexity scoring system was developed based on the natural acquisition timeline of syntax and morphology in children. Parts of speech were categorized by complexity level, with nouns and pronouns assigned to Level 1, as they are typically acquired first, around 10–12 months [64]. In contrast, more advanced grammatical structures, such as adjectives (including comparatives and superlatives), were assigned to Level 6, as their acquisition generally occurs later, around 5–6 years of age [64].

One challenge in classification involved particles and phrasal verbs, as there is limited research pinpointing the precise age at which they are acquired. While some phrasal verbs emerge early in language development [34], their intentional and contextual use may not become evident until later [52]. Another area requiring manual adjustment was the IN category, where “IN” is the tag used in Python’s NLTK package to denote both prepositions and subordinating conjunctions [5]. Because these word types develop at different times (prepositions around 27–30 months and subordinating conjunctions closer to 4 years of age) the research assistants manually assigned complexity levels based on their specific function within the sentence.

In the scoring system, points corresponded directly to complexity levels: Level 1 words received 1 point, Level 3 words received 3 points, and so forth. The full breakdown of the six-level system is presented in Table 2.

**Table 2:** Syntactic Complexity Scoring System Levels. Levels are listed by level number (Level), Part of speech abbreviation with written-out definition (Part of Speech), and stage of developmental acquisition (Acquired)

Level	Part of Speech	Acquired
1	NN—noun, singular	10–24 months
	NNP—proper noun, singular	12–24 months
	VB—verb, base form	16–20 months
2	VBG—verb, gerund/present participle	19–28 Months
	VBP—verb, sing. present	20 months
	JJ—adjective	20 months
	NNS – noun, plural	24–33 months
**3**	IN—prepositions	27–30 months
	POS—possessive ending	31–34 months
	CC—coordinating conjunction	36 months
	EX—existential there	36 months
	VBN—verb, past participle	40–48 months
	VBZ—verb, 3rd person sing. present	40–46 months
	VBD—verb, past tense	40–44—regular 40–46 months—irregular
4	WP—wh-pronoun	40 months
	DT—determiner	40–46 months
	WDT—wh-determiner	48 months
	RP—particle	First usage: 12–36 months. Understanding: 5–6 years Increase usage: 9 years
	PRP—personal pronoun	40 months
	PRP$—possessive pronoun	40–44- months
	MD—modal	40 months
5	CD—cardinal digit	3–4 years
	TO—to go	4–5 years
	IN—subordinating conjunctions	4 years
	JJR—adjective, comparative	5–7 years
6	RB—adverb	6–8 years
	RBS—adverb, superlative	6–8 years
	RBR—adverb, comparative	6–8 years

#### Individual Complexity Scores

To give individual syntactic complexity scores, part-of-speech data was input into an Excel document. Levels were color-coded, and the occurrence of each part of speech per level was calculated using an Excel formula. The total occurrence was multiplied by the level number to award the correct number of points associated with each level. The sum of the points was taken to obtain a total complexity score for individual sentences. These can be seen in Supplementary Table 2.

#### Validation of the Novel Complexity Scoring Method

The process to validate the syntax scoring system used in this paper included an independent analysis using the Systematic Analysis of Language Transcripts (SALT) software [40]. This validation process involved coding each sentence for syntactic features (i.e., all bound morphemes including affixes, suffixes, past/present tense verb endings, plural “s” marker, possessive “s,” third person verb “s,” as well as subordinate clauses, and syntactic errors). Then, each coded sentence was entered into SALT and analyzed. The analysis for each sentence generated a standard measures report. From these reports, MLU (mean length of utterance) in morphemes, number of verbs, number of different words, and the subordination index score were entered into a spreadsheet and added together to create a combined SALT results score. A correlation analysis compared the complexity scores derived using the original formula from this study and the combined SALT results score. Three Pearson's correlation analyses were run to compare the scores for (1) the sentences produced by the subjects in the ataxia group, (2) the sentences produced by the subjects in the control group, and (3) scores for all sentences produced by all subjects in the study. A high correlation between the complexity scores using the novel scoring method created for this study and the SALT results scores can provide validation of the original scoring method created for this study and confirm that it is an accurate measure of complexity.

Of note, the novel complexity scoring system utilized in this study was developed to obtain a nuanced understanding of expressive syntax. We anticipated that any differences in expressive language between groups might be sub-clinical, as no participants in the AD group were presenting with overt evidence of a language disorder based on clinical observation. Thus, it was critical to examine syntactic organization at a deeper level than can be obtained using the SALT program. Therefore, while the standard measures obtained with SALT were utilized to validate our complexity scoring system, the novel scoring method was necessary to address the goals of the study.

#### Speech Naturalness Ratings for Final Analysis

The final analysis evaluated the predictive factors of these complexity scores, in which speech naturalness was one of the predictors included in the model. Speech naturalness ratings were included as an exploratory predictor of syntactic complexity. Naturalness ratings, as commonly used in the perceptual evaluation of dysarthric speech, reflect an overall impression of how typical a speaker’s prosody, rate, and voice quality sound to a listener. We hypothesized that lower naturalness could be associated with reduced syntactic complexity. Specifically, speakers with severely disrupted prosody or strained vocal quality may simplify sentence structure as a compensatory mechanism to improve fluency or reduce the motor demands of speech. This inclusion allowed us to examine whether perceptual speech degradation corresponded with syntactic adaptation in ataxic dysarthria.

Speech naturalness was evaluated using a perceptual rating approach in which trained raters assessed speech samples on a seven-point interval scale, with higher scores indicating more natural-sounding speech. Raters were instructed to evaluate naturalness based on how well the sample adhered to expected norms of rate, rhythm, intonation, and stress patterning. Each speech sample was presented twice to the rater: first for naturalness scoring and then for orthographic transcription, ensuring that naturalness judgments were independent of intelligibility assessments. The ratings were completed by nine graduate students in speech-language pathology, all of whom were fluent in American English, had completed coursework on motor speech disorders, and reported normal hearing abilities. To ensure consistency, 15% of the trials were duplicated for intra-rater reliability analysis, and all trials were intensity-normalized to 70 dB. The same naturalness ratings used in this study were derived from the methodology detailed in Hilger, Cloud, and Fahey [23], which demonstrated moderate to high inter- and intra-rater reliability for speech naturalness assessments. For additional details on the rating procedures and methodology, refer to Hilger, Cloud, and Fahey [23].

#### Statistics

The goals of this analysis were fivefold: (1) to determine if the novel complexity score analysis was validated against SALT scoring, (2) to determine if there was a group difference in syntactic complexity across all sentences, (3) to determine if the inclusion of a dependent clause influenced syntactic complexity by group, (4) to determine if there was a group difference by the number of dependent clauses used, and (5) to analyze potential predictors within the ataxia group for syntactic complexity by dysarthria severity, dysarthria impact scoring, speech naturalness rating, age, and sex. For all goals apart from the fourth, linear mixed-effects regression models were built using the lme4 package [3] in RStudio (version 2022.07.1; [48]) running R (version 4.2.0; [50]). An alpha level of 0.05 was set as a reference for statistical significance using the lmertest package [33]. Cohen’s d was calculated for significant results using the effect size [4].

For the first goal, the complexity score was predicted by the interaction of the SALT score and group to determine if these two scoring systems correlated for all sentences combined as well as separately for the control vs. ataxia groups. For the second goal, the complexity score was predicted by group and whether the sentence had a dependent clause with random intercept included by participant. The dataset for this model included all sentences in the study. If the sentence included a dependent clause, it was labeled as “yes” in the binary dependent clause variable. For the third goal, we analyzed group differences for complexity scores for only independent clauses, and then only for dependent clauses. For the fourth goal, a generalized linear mixed-effects model (GLMM) was built using the lme4 package to determine if the number of dependent clauses was predicted by Group (Ataxia, Control) using a binomial GLMM, with random intercepts by Participant to account for repeated measures. Odds ratios and confidence intervals were computed to quantify the likelihood of producing dependent clauses by group. Finally, the fifth analysis used a linear mixed-effects regression model to determine if complexity score was predicted by age, sex, dysarthria severity, or speech naturalness rating with random intercept by participant.

## Results

### Validation of Complexity Score

**Fig. 1:**
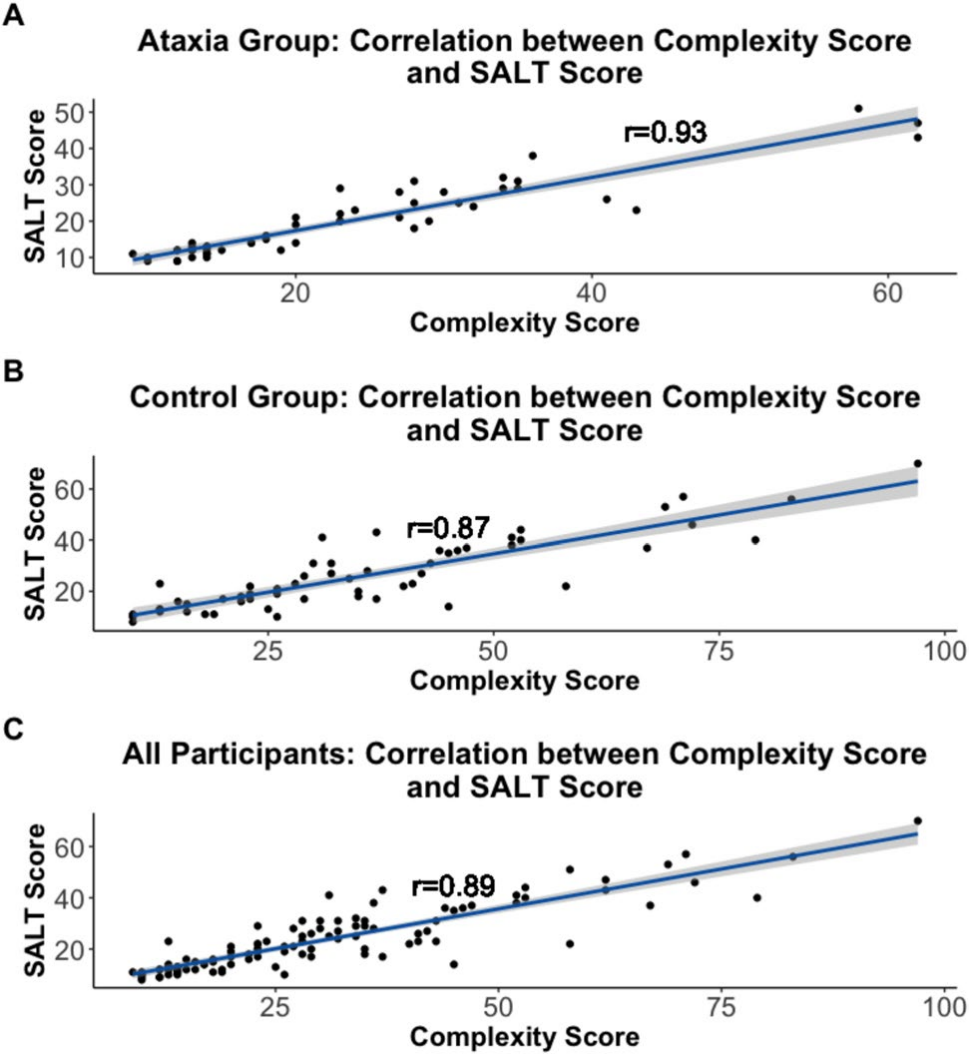
Correlation between novel sentence complexity scores and combination score using results from SALT analysis including (**a**) correlation between sentence complexity scores and SALT scores for the ataxia group, (**b**) correlation between sentence complexity scores and SALT scores for the control group, and (**c**) correlation between sentence complexity scores and SALT scores for sentences produced by both groups. The navy line shows the linear regression line between the two scores with standard error shown as the shaded grey region around the regression line

For the sentences produced by the participants in the ataxia group, there was a high correlation (*t*(50) = 17.91, p < 0.0001, r = 0.93) between the complexity scores derived using the original formula created for this study and the combined SALT results score (see Fig. 1A). For the sentences produced by the subjects in the control group, there was also a high correlation.

(*t*(54) = 13.10, p < 0.0001, r = 0.87) between the complexity scores derived using the original formula and the combined SALT results score (see Fig. 1B). Finally, the correlation between the complexity scores derived using the original formula and the combined SALT results score for all participants in the study was also high (*t*(106) = 20.47, p < 0.0001, r = 0.89) (see Fig. 1C). Overall, the high correlation between the complexity scores using the original formula and the combined SALT scores demonstrates high validity for this scoring system.

### Group Comparison Across All Sentence Types

**Fig. 2:**
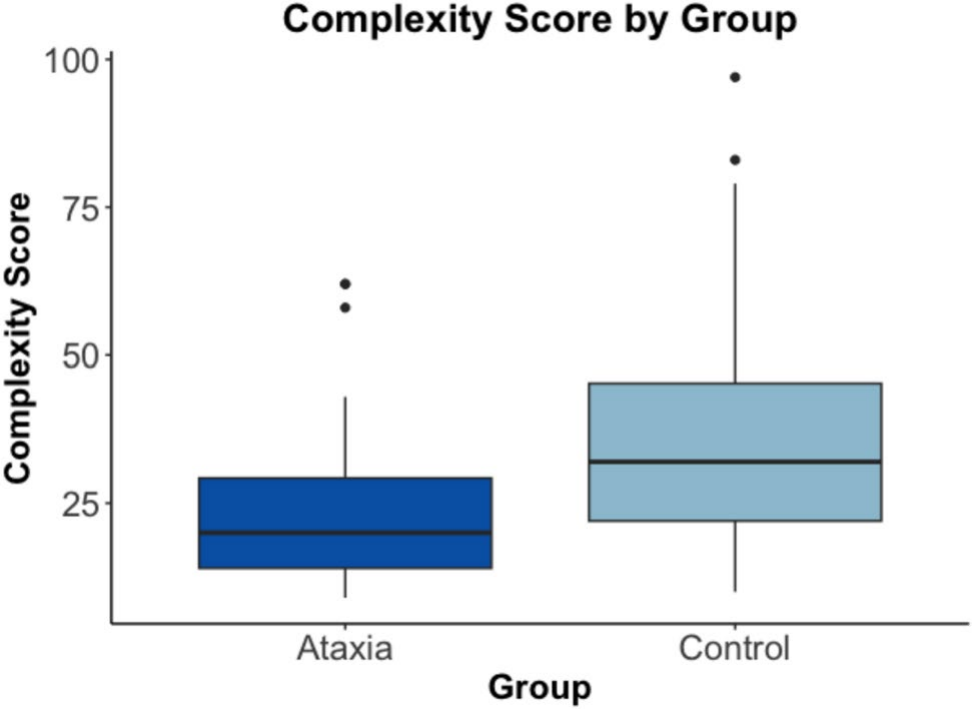
Box and whiskers plot of the complexity score by group. The box and whiskers plot shows the median pitch complexity score (middle dark line), the interquartile range (outline of the box), and values outside of the interquartile range (dots)

Figure 2 displays the group differences for syntactic complexity across all sentences in the study with values provided in Table 3. Across all sentences, the control group had higher complexity scores (mean = 36.18, SD = 20.15) than the group with ataxia (mean = 23.46, SD = 12.78). Even though this difference in complexity scores is not considered statistically significant according to the p-value, there was still a small effect using Cohen’s D (*t*(70.687) = 1.93, p = 0.057, Cohen’s D = 0.39). Therefore, the control speakers did produce more syntactically complex utterances, but this was a small effect that was not statistically significant. For both groups of speakers, sentences with a dependent clause were more syntactically complex than sentences without dependent clauses (*t*(103.60) = 2.72, p = 0.008, Cohen’s d = 0.74).

**Table 3:** Group Complexity Score. Group complexity score is listed by clause type (Independent, Dependent, Overall (Independent + Dependent), group type (Control or AD), and data type (Total Score or Average Score)

	Independent Clauses	Dependent Clauses	Overall Complexity Score
Control Group Total	1737	922	2588
AD Group Total	1240	427	1667
Control Group Average	62.04	30.73	92.43
AD Group Average	47.69	16.42	64.12

### Group Differences in Independent Clause Complexity

**Fig. 3:**
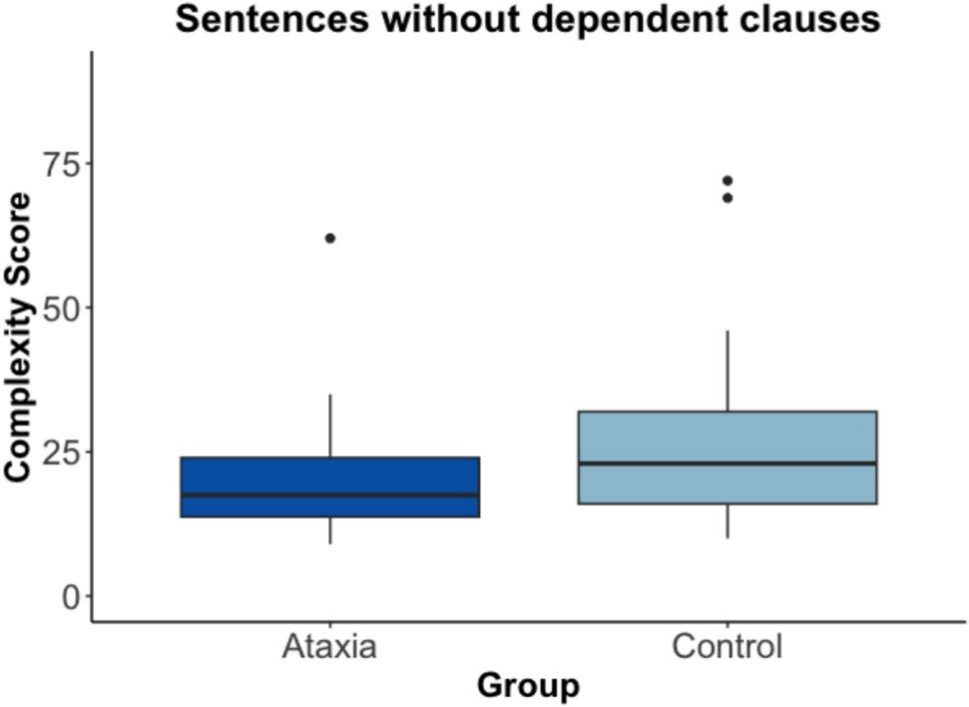
Box plot of complexity scores by group for the independent clauses only. The box and whiskers plot shows the median pitch complexity score (middle dark line), the interquartile range (outline of the box), and values outside of the interquartile range (dots)

To further analyze independent clause complexity, sentences with dependent clauses were removed from the dataset. A significant group difference was seen with a medium effect size (*t*(36.61) = 2.28, p = 0.03, Cohen’s d = 0.65). As shown in Fig. 3, for sentences without dependent clauses, control speakers had higher syntactic complexity (mean = 27.06, SD = 15.21) compared to the speakers with ataxia (mean = 20.30, SD = 10.10).

### Group Differences in Dependent Clause Complexity

**Fig. 4:**
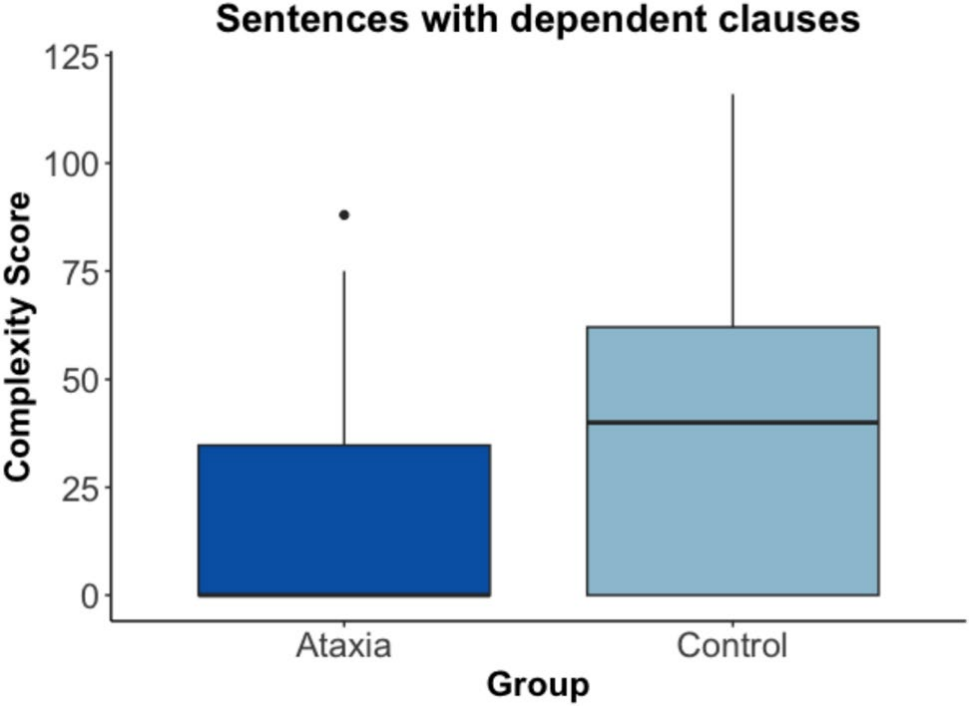
Box plot of complexity score by group for dependent clauses only. The box and whiskers plot shows the median pitch complexity score (middle dark line), the interquartile range (outline of the box), and values outside of the interquartile range (dots)

When analyzing the complexity of dependent clauses between the two groups, the results show that dependent clause complexity was significantly different by group, (*t*(52) = 2.65, p = 0.01, Cohen’s d = 0.68). As shown in Fig. 4, for dependent clauses, control speakers had higher syntactic complexity (mean = 43.79, SD = 37.88) compared to the speakers with ataxia (mean = 19.88, SD = 27.19).

### Group Differences in the Use of Dependent Clauses

To examine whether the number of dependent clauses significantly differed by group, a generalized linear mixed model (GLMM) was fit using a binomial family with Group (Ataxia, Control) as the predictor and Participant as a random effect. Results indicated a significant effect of Group on the likelihood of producing dependent clauses (β = 0.99, SE = 0.44, z = 2.26, p = 0.024), suggesting that control speakers produced significantly more dependent clauses than individuals with AD. The odds ratio (OR = 2.69) indicated that control speakers were 2.69 times more likely to use dependent clauses compared to speakers with AD.

### Syntactic Complexity within the Ataxia Speakers

The final analysis assessed whether syntactic complexity was predicted by factors within the ataxia speakers, including dysarthria severity, age, sex, speech naturalness, and Dysarthria Impact Profile score. There was no significant difference seen for any of the predicting factors (p > 0.05). Specifically, there were no relationships between dysarthria severity, age, sex, speech naturalness, and Dysarthria Impact Profile score, and syntactic complexity.

## Discussion

This study aimed to investigate whether syntactic complexity differs between individuals with AD and healthy controls, and if so, whether any observed reduction is more consistent with the effects of cerebellar impairment or with an adaptive adjustment to reduce speech-motor demands. Consistent with our research questions, we investigated (1) whether syntactic complexity differs between individuals with ataxic dysarthria and control speakers, (2) if differences were observed, whether they might reflect underlying cerebellar damage or potential adaptive strategies to manage speech-motor demands, and (3) whether demographic or disease-related factors such as age, sex, dysarthria severity, speech naturalness rating, or dysarthria impact scoring were associated with syntactic complexity.

A novel syntactic complexity scoring system was developed to provide a structured and objective measure of syntactic variation in speakers with AD. This system was designed based on the natural acquisition timeline of syntax and morphology, categorizing parts of speech by complexity level. Given the lack of standardized tools that specifically assess syntactic complexity in disordered speech in adults, this method was developed to fill that gap and offer a more precise analysis of syntactic structures. This novel scoring system was validated using the SALT software tool, a tool commonly used in research and clinical practice for analyzing syntactic features and complexity (e.g., [9, 31, 36, 43, 60]). The results from all correlation analyses confirmed the validity of the novel method for scoring sentence complexity used in this study.

Findings from this study suggest that speakers with AD demonstrate reduced syntactic complexity compared to controls, particularly in the use of dependent clauses. However, no significant correlations were found between syntactic complexity and demographic or disease-related variables (i.e., age, sex, dysarthria severity, speech naturalness rating, dysarthria impact scoring). If syntactic simplification was a compensatory strategy for motor speech impairment, we would expect some degree of correlation to emerge between syntactic complexity and dysarthria severity and/or impact; yet such a pattern was not observed here. We would also not expect differences in syntactic production across both independent and dependent clauses if speakers were simplifying their language solely as a compensatory strategy, as such simplification would more likely be limited to dependent clauses, which are typically more complex. Thus, it appears most likely that our findings reflect a fundamental change in linguistic structuring due to cerebellar dysfunction. Additionally, the lack of correlation between syntactic complexity and age or sex indicates that these reductions occur across demographic groups, further supporting the notion that syntactic impairment in AD is neurologically driven rather than influenced by social or age factors.

These findings align with previous work indicating that cerebellar damage can affect expressive syntax [6, 28] and contribute to non-motor linguistic deficits [37, 56], as well as with clinical evidence from cerebellar stroke populations. For example, Satoer et al. [55] identified mild but statistically significant language deficits across multiple linguistic domains, including syntax, in patients with cerebellar lesions, even in the absence of cortical involvement. Notably, their findings showed similar syntactic and semantic disruptions as seen in cerebral aphasia, reinforcing the cerebellum’s involvement in higher-order language functions. This complements our findings that syntactic simplification in AD is not clearly explained by motor severity or compensatory behavior, but may reflect a direct linguistic impairment.

Moreover, the finding that speech naturalness did not predict syntactic complexity suggests that reduced naturalness in speech does not necessarily accompany syntactic reductions in AD. If syntax were simplified as a compensatory strategy to improve speech fluency or clarity, we might expect a correlation between more unnatural-sounding speech and greater syntactic reduction. Instead, our results indicate that speakers with less natural-sounding speech did not necessarily produce simpler syntax than those with more natural-sounding speech. This finding reinforces the argument that syntactic simplification is not an intentional adaptation to improve intelligibility or fluency but rather likely an intrinsic linguistic characteristic of cerebellar ataxia.

The observed reduction in syntactic complexity, regardless of dysarthria severity or impact, further supports the conclusion that cerebellar impairment contributes to expressive syntactic deficits. If syntactic simplification were a compensatory behavior, we would expect greater syntactic reduction in individuals with more severe dysarthria and/or in those experiencing greater psychosocial impact from their dysarthria. However, since syntactic complexity remained independent of dysarthria severity or Dysarthria Impact Profile scores, it suggests that cerebellar dysfunction may directly impact syntactic formulation, independent of speech-motor difficulties.

A more detailed analysis of sentence structure revealed additional insights. Individuals with AD used fewer dependent clauses overall, and when producing independent clauses, their syntactic structures were significantly less complex than those of control speakers. This trend was consistent across all sentence types, suggesting a general difference in hierarchical syntactic organization. These findings align with prior research demonstrating that clausal structure is closely linked to syntactic complexity [64]

Although the absence of a correlation between syntactic complexity and speech-motor variables suggests the reductions may not be purely compensatory, we acknowledge this evidence is indirect. It remains possible that speakers with AD adopted an economy of words strategy to reduce production effort. Future studies should incorporate task manipulations or dual-task paradigms to better distinguish linguistic impairment from adaptive strategies.

An important observation in our dataset is the considerable variability in syntactic complexity scores among individuals with ataxic dysarthria. This heterogeneity likely reflects the broad clinical diversity seen in cerebellar disorders, which can vary by etiology (e.g., hereditary vs. acquired), disease stage, rate of progression, and the extent of extracerebellar involvement. While our exploratory analyses included several speech-related measures (e.g., dysarthria severity, naturalness, impact), these factors only partially accounted for the observed variation. Other contributors may include individual differences in linguistic ability, educational background, and the use of unconscious compensatory strategies. We acknowledge that the relatively small sample size limited our ability to conduct more fine-grained subgroup or interaction analyses. Future studies with larger and more clinically stratified cohorts will be better positioned to examine the sources of intra-group variability and to isolate more precisely the language correlates of cerebellar involvement.

### Clinical Implications

Through this analysis, we have identified a reduction in syntactic complexity in individuals with AD, yet its precise etiology remains uncertain. Our preliminary findings support the notion that cerebellar dysfunction impacts expressive syntax, and this insight can inform potential therapy approaches for individuals with AD. It is established that therapy targeting compensatory strategies for rate, prosody, and phonation can support speakers with AD as they navigate fatigue and speech difficulties [35, 65]. It is plausible that explicit instruction in the domain of syntactic complexity may support additional improvement in communicative success. Intervention may include education related to syntax and its attributes that contribute to linguistic complexity. After being provided with education on syntactic functions, a client may be interested in targeting goals to intentionally increase syntactic complexity or purposefully reduce complexity during differing communicative interactions. Our current findings could expand targets for speech therapy that might improve communicative confidence and participation, which in turn, can increase quality of life among individuals with cerebellar ataxia.

### Limitations

One limitation of this study is the exclusive use of a picture description task to assess syntactic complexity. While this task provides a structured yet naturalistic way to elicit connected speech, it may not fully capture the range of syntactic abilities individuals with AD use across different communicative contexts. Different speaking tasks, such as conversational discourse, narrative storytelling, or procedural descriptions, may elicit varying levels of syntactic complexity and place different cognitive and motor demands on speakers. For example, spontaneous conversation may lead to more simplified syntax due to real-time processing demands, while structured storytelling could encourage greater syntactic elaboration. By relying solely on picture description, the findings may reflect task-specific syntactic patterns rather than a generalized syntactic deficit in AD. Future research should incorporate multiple speech-elicitation tasks to determine whether syntactic simplification in AD is consistent across contexts or varies depending on communicative demands and cognitive load.

Another limitation of this study is the limited number of sentences analyzed per participant. We selected two complete sentences per individual to ensure manual verification of sentence completeness and consistency in applying the novel syntactic scoring system. While this small sample may not fully reflect each speaker’s maximal syntactic ability, our goal was to detect group-level trends in naturally produced speech under controlled conditions. The high correlation between our complexity scores and standard SALT metrics supports the validity of this approach. Nonetheless, we acknowledge that a broader sampling of each participant’s language could provide a more comprehensive view of their syntactic range. Future research will expand the number of utterances analyzed and incorporate diverse speech tasks to assess intra-speaker variability and expressive potential more fully.

We also recognize that the allotted time for the picture description task (2–3 min) may not have been sufficient for all individuals with motor speech disorders to fully express their syntactic repertoire. While participants were not cut off, future studies may benefit from explicitly tailoring task durations to accommodate individual motor speech profiles.

Another key limitation concerns the underlying neurological profiles of the participants in the ataxia group. While all participants had confirmed diagnoses of cerebellar ataxia, most were living with progressive hereditary conditions (e.g., SCA subtypes, Friedreich’s Ataxia) that are known to involve widespread neural degeneration beyond the cerebellum, including spinal tracts and cortical structures. We did not collect neuroimaging data or conduct subtype-specific analyses to verify the localization of pathology. As such, we cannot rule out the possibility that reduced syntactic complexity reflects more diffuse neurodegeneration rather than cerebellar involvement alone. Future studies should incorporate neuroimaging, disease staging, and more detailed etiological classification to better isolate the cerebellum’s contribution to language outcomes. A further limitation is the absence of detailed etiological data, including time since onset, and measures of disease progression. While our focus was on speech-related variables that could be consistently coded across participants, future studies should incorporate clinical and neuroimaging markers to better account for variability in syntactic output associated with broader patterns of neurodegeneration.

## Conclusion

This study examined syntactic complexity in individuals with ataxic dysarthria (AD) compared to healthy controls, with a focus on the use of dependent clauses. While a trend toward reduced syntactic complexity was observed in the AD group,particularly in the use of dependent clauses,this pattern did not reach statistical significance, and there was considerable variability across participants. Furthermore, syntactic complexity did not significantly correlate with speech-related variables such as dysarthria severity, speech naturalness, or self-perceived communication impact, nor with demographic factors such as age or sex. These findings suggest that syntactic simplification may not be solely attributable to compensatory adaptations to speech-motor demands. However, they do not offer conclusive evidence of a specific linguistic impairment tied to cerebellar dysfunction. Instead, the results point to the complex and multifactorial nature of expressive language in AD, likely influenced by both motor and cognitive-linguistic factors.

This study contributes to an evolving literature on the cerebellum’s broader role in language, while also underscoring the importance of interpreting group trends with caution given the individual variability observed. Future research should include a wider range of speaking tasks, more extensive sampling per participant, and neuroimaging measures to better characterize the mechanisms underlying syntactic performance in cerebellar disorders. Clinically, the findings support the potential value of assessing expressive language in AD as part of comprehensive management, particularly for individuals who may benefit from language-based supports in addition to motor-focused interventions.

## Supplementary Information

Below is the link to the electronic supplementary material.

Supplementary file1 (DOCX 21 KB)

## Data Availability

Data is provided within the manuscript or supplementary information files.

## References

[1] Ackermann H, Mathiak K, Riecker A. The contribution of the cerebellum to speech production and speech perception: Clinical and functional imaging data. Cerebellum (London, England). 2007;6(3):202–13. 10.1080/14734220701266742. 17786816 10.1080/14734220701266742

[2] Baldo JV, Dronkers NF. Neural correlates of arithmetic and language comprehension: a common substrate? Neuropsychologia. 2007;45(2):229–35. 10.1016/j.neuropsychologia.2006.07.014. 16997333 10.1016/j.neuropsychologia.2006.07.014

[3] Bates D, Maechler M, Bolker B, Walker S. lme4: Linear mixed-effects models using Eigen and S4. R Package Version. 2014;1(7):1–23.

[4] Ben-Shachar MS, Lüdecke D, Makowski D. effectsize: Estimation of effect size indices and standardized parameters. J Open Source Softw. 2020;5(56):2815.

[5] Bird S, Klein E, Loper E. Natural Language processing with python: analyzing text with the natural language toolkit. Sebastopol, CA: O’Reilly Media, Inc. 2009.

[6] Bodranghien F, Bastian A, Casali C, Hallett M, Louis ED, Manto M, Mariën P, Nowak DA, Schmahmann JD, Serrao M, Steiner KM, Strupp M, Tilikete C, Timmann D, van Dun K. Consensus paper: revisiting the symptoms and signs of cerebellar syndrome. The Cerebellum. 2016;15(3):369–91. 10.1007/s12311-015-0687-3. 26105056 10.1007/s12311-015-0687-3 PMC5565264

[7] Bohland JW, Guenther FH. An fMRI investigation of syllable sequence production. Neuroimage. 2006;32(2):821–41. 10.1016/j.neuroimage.2006.04.173. 16730195 10.1016/j.neuroimage.2006.04.173

[8] Brennan J, Nir Y, Hasson U, Malach R, Heeger DJ, Pylkkänen L. Syntactic structure building in the anterior temporal lobe during natural story listening. Brain Lang. 2012;120(2):163–73. 10.1016/j.bandl.2010.04.002. 20472279 10.1016/j.bandl.2010.04.002 PMC2947556

[9] Castilla-Earls A, Fulcher-Rood K. Convergent and divergent validity of the grammaticality and utterance length instrument. J Speech Lang Hear Res. 2018;61(1):120–9. 10.1044/2017_JSLHR-L-17-0152. 29346497 10.1044/2017_JSLHR-L-17-0152

[10] Chen SHA, Desmond JE. Cerebrocerebellar networks during articulatory rehearsal and verbal working memory tasks. Neuroimage. 2005;24(2):332–8. 10.1016/j.neuroimage.2004.08.032. 15627576 10.1016/j.neuroimage.2004.08.032

[11] De Smet HJ, Baillieux H, De Deyn PP, Mariën P, Paquier P. The cerebellum and language: the story so far. Folia Phoniatr Logop. 2007;59(4):165–70. 10.1159/000102927. 17627124 10.1159/000102927

[12] Dronkers N, Ogar J. Brain areas involved in speech production. Brain. 2004;127(7):1461–2. 10.1093/brain/awh233. 15197111 10.1093/brain/awh233

[13] Enderby, P. M., & Palmer, R. Frenchay Dysarthria Assessment: Examiner's Manual, Pro. ed. 2008.

[14] Esterman M, Thai M, Okabe H, DeGutis J, Saad E, Laganiere SE, Halko MA. Network-targeted cerebellar transcranial magnetic stimulation improves attentional control. Neuroimage. 2017;156:190–8. 10.1016/j.neuroimage.2017.05.011. 28495634 10.1016/j.neuroimage.2017.05.011 PMC5973536

[15] Evers S. The cerebellum in musicology: a narrative review. The Cerebellum. 2024;23(3):1165–75. 10.1007/s12311-023-01594-6. 37594626 10.1007/s12311-023-01594-6 PMC11102367

[16] Evers S, Tölgyesi B (2022) Music and the cerebellum. In The Emotional Cerebellum pp. 195–212. Springer, Cham. 10.1007/978-3-030-99550-8_13 10.1007/978-3-030-99550-8_13 35902473

[17] Fiez J, Raife E, Balota D, Schwarz J, Raichle M, Petersen S. A positron emission tomography study of the short-term maintenance of verbal information. J Neurosci. 1996;16(2):808–22. 10.1523/JNEUROSCI.16-02-00808.1996. 8551361 10.1523/JNEUROSCI.16-02-00808.1996 PMC6578642

[18] Friederici AD. Language in our brain: the origins of a uniquely human capacity. Cambridge, MA: MIT Press. 2017.

[19] Goodglass H. Understanding aphasia. Academic Press; 1993.

[20] Goodglass H, Wingfield A, Hyde MR, Theurkauf JC. Category specific dissociations in naming and recognition by aphasic patients. Cortex. 1986;22(1):87–102. 10.1016/S0010-9452(86)80034-X. 2423298 10.1016/s0010-9452(86)80034-x

[21] Grodzinsky Y, Santi A. The battle for Broca’s region. Trends Cogn Sci. 2008;12(12):474–80. 10.1016/j.tics.2008.09.001. 18930695 10.1016/j.tics.2008.09.001

[22] Guell X, Gabrieli JDE, Schmahmann JD. Triple representation of language, working memory, social and emotion processing in the cerebellum: convergent evidence from task and seed-based resting-state fMRI analyses in a single large cohort. Neuroimage. 2018;172:437–49. 10.1016/j.neuroimage.2018.01.082. 29408539 10.1016/j.neuroimage.2018.01.082 PMC5910233

[23] Hilger A, Cloud C, Fahey T. Speech impairment in cerebellar ataxia affects naturalness more than intelligibility. The Cerebellum. 2022. 10.1007/s12311-022-01427-y. 35670895 10.1007/s12311-022-01427-y

[24] Hilger A, Dunne-Platero K (2022) The experiences of speech pathology referral and communicative participation in adults with cerebellar ataxia. Int J Speech-Language Pathol 0(0):1–12. 10.1080/17549507.2022.2134455 10.1080/17549507.2022.2134455 36562755

[25] Hilger AI. Impaired sensorimotor integration for prosodic production in Ataxic Dysarthria. (Doctoral dissertation, Northwestern University). 2020.

[26] Hux K, Frodsham K. Speech and language characteristics of neurologically healthy adults when describing the modern cookie theft picture: mixing the new with the old. Am J Speech Lang Pathol. 2023;32(3):1110–30. 36898138 10.1044/2022_AJSLP-22-00291

[27] Ivry RB, Spencer RM, Zelaznik HN, Diedrichsen J. The cerebellum and event timing. Ann N Y Acad Sci. 2002;978(1):302–17. 10.1111/j.1749-6632.2002.tb07576.x. 12582062 10.1111/j.1749-6632.2002.tb07576.x

[28] Justus T. The cerebellum and english grammatical morphology: evidence from production, comprehension, and grammaticality judgments. J Cogn Neurosci. 2004;16(7):1115–30. 10.1162/0898929041920513. 15453968 10.1162/0898929041920513 PMC2811412

[29] Kent RD, Kent JF, Duffy JR, Thomas JE, Weismer G, Stuntebeck S. Ataxic dysarthria. J Speech Lang Hear Res. 2000;43(1–5):1275–89. 10.1044/jslhr.4305.1275. 11063247 10.1044/jslhr.4305.1275

[30] Kent RD, Netsell R, Abbs JH. Acoustic characteristics of dysarthria associated with cerebellar disease. J Speech Hear Res. 1979;22(3):627–48. 10.1044/jshr.2203.627. 502519 10.1044/jshr.2203.627

[31] King D, Palikara O. Assessing language skills in adolescents with autism spectrum disorder. Child Language Teach Ther. 2018;34(2):101–13. 10.1177/0265659018780968.

[32] Koziol LF, Budding D, Andreasen N, D’Arrigo S, Bulgheroni S, Imamizu H, Ito M, Manto M, Marvel C, Parker K, Pezzulo G, Ramnani N, Riva D, Schmahmann J, Vandervert L, Yamazaki T. Consensus paper: the cerebellum’s role in movement and cognition. The Cerebellum. 2014;13(1):151–77. 10.1007/s12311-013-0511-x. 23996631 10.1007/s12311-013-0511-x PMC4089997

[33] Kuznetsova A, Brockhoff PB, Christensen RHB (2017) lmertest package: tests in linear mixed effects models. J Stat Softw 82(13). 10.18637/jss.v082.i13

[34] Lodge DN, Leach EA. Children’s acquisition of idioms in the english language. J Speech Hear Res. 1975;18(3):521–9. 10.1044/jshr.1803.521. 1186161 10.1044/jshr.1803.521

[35] Lowit A, Egan A, Hadjivassilliou M (2019, November 14) Speech treatment for people with hereditary ataxia – a feasibility study. International Ataxia Research Conference (IARC) 2019. International Ataxia Research Conference (IARC) 2019, USA. https://strathprints.strath.ac.uk/71465/

[36] Lundine JP, Harnish SM, McCauley RJ, Zezinka AB, Blackett DS, Fox RA. Exploring summarization differences for two types of expository discourse in adolescents with traumatic brain injury. Am J Speech Lang Pathol. 2018;27(1):247–57. 10.1044/2017_AJSLP-16-0131. 29121200 10.1044/2017_AJSLP-16-0131

[37] Mariën P, Ackermann H, Adamaszek M, Barwood CHS, Beaton A, Desmond J, De Witte E, Fawcett AJ, Hertrich I, Küper M, Leggio M, Marvel C, Molinari M, Murdoch BE, Nicolson RI, Schmahmann JD, Stoodley CJ, Thürling M, Timmann D, … Ziegler W (2014) Consensus paper: language and the cerebellum: an ongoing enigma. Cerebellum 13(3):386–410. 10.1007/s12311-013-0540-5 10.1007/s12311-013-0540-5 PMC4090012 24318484

[38] Marvel CL, Desmond JE. The contributions of cerebro-cerebellar circuitry to executive verbal working memory. Cortex. 2010;46(7):880–95. 10.1016/j.cortex.2009.08.017. 19811779 10.1016/j.cortex.2009.08.017 PMC2872048

[39] Matchin W, Hickok G. The cortical organization of syntax. Cereb Cortex. 2020;30(3):1481–98. 31670779 10.1093/cercor/bhz180 PMC7132936

[40] Miller J, Chapman R. SALT: Systematic analysis of language transcripts. Madison, WI: University of Wisconsin; 1993.

[41] Nasreddine ZS, Phillips NA, Bédirian V, Charbonneau S, Whitehead V, Collin I, ... Chertkow H. The Montreal Cognitive Assessment, MoCA: a brief screening tool for mild cognitive impairment. J Am Geriatr Soc. 2005;53(4):695–699 10.1111/j.1532-5415.2005.53221.x 15817019

[42] National Ataxia Foundation (n.d.) National Ataxia Foundation. (n.d.). https://www.ataxia.org

[43] Nippold MA, Frantz-Kaspar MW, Vigeland LM. Spoken language production in young adults: examining syntactic complexity. J Speech Lang Hear Res. 2017;60(5):1339–47. 10.1044/2016_JSLHR-L-16-0124. 28492843 10.1044/2016_JSLHR-L-16-0124

[44] Pastor MA, Day BL, Macaluso E, Friston KJ, Frackowiak RSJ. The functional neuroanatomy of temporal discrimination. J Neurosci. 2004;24(10):2585–91. 10.1523/JNEUROSCI.4210-03.2004. 15014134 10.1523/JNEUROSCI.4210-03.2004 PMC6729480

[45] Paul R, Norbury C, Gosse C. Language disorders: listening, speaking. Reading, writing, and communicating. 5th ed. St. Louis, MO: Mosby. 2017.

[46] Peelle JE, Troiani V, Gee J, Moore P, McMillan C, Vesely L, Grossman M. Sentence comprehension and voxel-based morphometry in progressive nonfluent aphasia, semantic dementia, and nonaphasic frontotemporal dementia. J Neurolinguistics. 2008;21(5):418–32. 10.1016/j.jneuroling.2008.01.004. 19727332 10.1016/j.jneuroling.2008.01.004 PMC2598754

[47] Pillay SB, Binder JR, Humphries C, Gross WL, Book DS. Lesion localization of speech comprehension deficits in chronic aphasia. Neurology. 2017;88(10):970–5. 10.1212/WNL.0000000000003683. 28179469 10.1212/WNL.0000000000003683 PMC5333516

[48] Posit team (2022) RStudio: Integrated Development Environment for R [Computer software]. Posit Software, PBC. http://www.posit.co/

[49] Prati JM, Pontes-Silva A, Gianlorenço ACL. The cerebellum and its connections to other brain structures involved in motor and non-motor functions: a comprehensive review. Behav Brain Res. 2024;465:114933. 10.1016/j.bbr.2024.114933. 38458437 10.1016/j.bbr.2024.114933

[50] R Core Team (2022) R: A language and environment for statistical computing [Computer software]. R Foundation for Statistical Computing. https://www.R-project.org/.

[51] Reilly J, Fisher JL. Sherlock holmes and the strange case of the missing attribution: a historical note on “The Grandfather Passage.” J Speech Lang Hear Res. 2012;55(1):84–8. 10.1044/1092-4388(2011/11-0158). 22354714 10.1044/1092-4388(2011/11-0158)

[52] Riguel E (2015) Les phrasal verbs: usage et acquisition. Textes & Contextes, 9, http://preo.u.

[53] Rogalsky C, LaCroix AN, Chen K-H, Anderson SW, Damasio H, Love T, Hickok G. The neurobiology of agrammatic sentence comprehension: a lesion study. J Cogn Neurosci. 2018;30(2):234–55. 10.1162/jocn_a_01200. 29064339 10.1162/jocn_a_01200 PMC6434535

[54] Sandford Health. *Rare Disease Registry*. Sanford Health. (n.d.). Retrieved July7, 2025 from https://research.sanfordhealth.org/rare-disease-registry

[55] Satoer D, Koudstaal PJ, Visch-Brink E, van der Giessen RS. Cerebellar-induced aphasia after stroke: evidence for the “Linguistic Cerebellum.” The Cerebellum. 2024;23(4):1457–65. 10.1007/s12311-024-01658-1. 38244134 10.1007/s12311-024-01658-1 PMC11269354

[56] Schmahmann JD. The cerebellum and cognition. Neurosci Lett. 2019;688:62–75. 10.1016/J.NEULET.2018.07.005. 29997061 10.1016/j.neulet.2018.07.005

[57] Schmahmann JD, Sherman JC. The cerebellar cognitive affective syndrome. Brain. 1998;121(4):561–79. 10.1093/brain/121.4.561. 9577385 10.1093/brain/121.4.561

[58] Schweizer TA, Alexander MP, Susan Gillingham BA, Cusimano M, Stuss DT. Lateralized cerebellar contributions to word generation: a phonemic and semantic fluency study. Behav Neurol. 2010;23(1–2):31–7. 10.3233/BEN-2010-0269. 20714059 10.3233/BEN-2010-0269 PMC5434417

[59] Shekari E, Nozari N. A narrative review of the anatomy and function of the white matter tracts in language production and comprehension. Front Hum Neurosci. 2023;17:1139292 10.3389/fnhum.2023.1139292 PMC10083342 37051488

[60] Shivabasappa P, Peña ED, Bedore LM. Typicality effect and category structure in spanish-english bilingual children and adults. J Speech Lang Hear Res. 2017;60(6):1577–89. 10.1044/2016_JSLHR-L-15-0377. 28535193 10.1044/2016_JSLHR-L-15-0377 PMC5544412

[61] Spencer KA, Slocomb DL. The neural basis of ataxic dysarthria. The Cerebellum. 2007;6:58–65. 10.1080/14734220601145459. 17366266 10.1080/14734220601145459

[62] Stoodley CJ, Schmahmann JD. Evidence for topographic organization in the cerebellum of motor control versus cognitive and affective processing. Cortex. 2010;46(7):831–44. 10.1016/j.cortex.2009.11.008. 20152963 10.1016/j.cortex.2009.11.008 PMC2873095

[63] Strick PL, Dum RP, Fiez JA. Cerebellum and Nonmotor Function. Annual Review of Neuroscience. 2009;32(2009):413–34. 10.1146/annurev.neuro.31.060407.125606. 19555291 10.1146/annurev.neuro.31.060407.125606

[64] Turnbull K, Justice L (2017) Language Development From Theory to Practice. https://www.pearson.com/en-us/subject-catalog/p/language-development-from-theory-to-practice/P200000001676/9780134170671

[65] Vogel AP, Graf LH, Magee M, Schöls L, Rommel N, Synofzik M. Home-based biofeedback speech treatment improves dysarthria in repeat-expansion SCAs. Ann Clin Transl Neurol. 2022;9(8):1310–5. 10.1002/acn3.51613. 35726838 10.1002/acn3.51613 PMC9380135

[66] Walshe M, Peach RK, Miller N. Dysarthria Impact Profile: Development of a scale to measure psychosocial effects. Int J Lang Commun Disord. 2009;44(5):693–715. 10.1080/13682820802317536. 18821230 10.1080/13682820802317536

[67] Wilson SM, Saygın AP. Grammaticality judgment in Aphasia: deficits are not specific to syntactic structures, aphasic syndromes, or lesion sites. J Cogn Neurosci. 2004;16(2):238–52. 10.1162/089892904322984535. 15068594 10.1162/089892904322984535

[68] Wulfeck B, Bates E. Differential sensitivity to errors of agreement and word order in Broca’s Aphasia. J Cogn Neurosci. 1991;3(3):258–72. 10.1162/jocn.1991.3.3.258. 23964841 10.1162/jocn.1991.3.3.258

[69] Yorkston KM, Beukelman DR. Ataxic dysarthria: Treatment sequences based on intelligibility and prosodic considerations. J Speech Hearing Disord. 1981;46(4):398–404. 10.1044/jshd.4604.398. 7300267 10.1044/jshd.4604.398

