# Supplementary material for: Revisiting the Cerebellum's Linguistic Role: Evidence for Cerebellar Involvement in Expressive Syntax
Source: Cerebellum. 2025 Jul 10;24(5):126. doi: 10.1007/s12311-025-01879-y (PMC12245948; doi:10.1007/s12311-025-01879-y)
Supplement: Supplementary file 1 — Supplementary file1 (DOCX 21 KB) [file 12311_2025_1879_MOESM1_ESM.docx]

**Supplemental Table 1:** Part of Speech Occurrences. Parts of Speech are listed by their Python script abbreviation and its written-out definition, the number of POS in control samples (Control Count), and the number of POS in ataxic dysarthria samples (AD Count).

| Part of Speech | Control Count | AD Count |
| --- | --- | --- |
| NN - noun, singular | 160 | 114 |
| NNP - proper noun, singular | 1 | 0 |
| VB - verb, base form | 177 | 125 |
| VBG - verb, gerund/present participle | 63 | 42 |
| VBP - verb, sing. present | 15 | 10 |
| JJ - adjective | 23 | 5 |
| NNS – noun, plural | 34 | 28 |
| IN – prepositions/ subordinating conjunctions | 89 | 50 |
| POS - possessive ending | 2 | 0 |
| CC - coordinating conjunction | 16 | 13 |
| EX - existential there | 4 | 2 |
| VBN - verb, past participle | 3 | 1 |
| VBZ - verb, 3rd person sing. present | 68 | 46 |
| DT - determiner | 114 | 73 |
| VBD - verb, past tense | 6 | 7 |
| WP - wh-pronoun | 5 | 1 |
| WDT - wh-determiner | 2 | 1 |
| RP - particle | 52 | 41 |
| PRP - personal pronoun | 39 | 32 |
| PRP$ - possessive pronoun | 7 | 8 |
| MD - modal | 1 | 2 |
| CD - cardinal digit | 4 | 2 |
| TO - to go | 20 | 13 |
| JJR - adjective, comparative | 2 | 0 |
| RB - adverb | 17 | 9 |
| RBS - adverb, superlative | 1 | 0 |
| RBR - adverb, comparative | 1 | 0 |

**Supplementary Table 2:** Complexity Score by Sentence. Sentence complexity score is listed by participant number (Participant), total sentence complexity score (Complexity Score), presence of dependent clause (DEP = Dependent Clause), group type (Group = Ataxia or Control).

| Participant | Complexity score | DEP | Group |
| --- | --- | --- | --- |
| A01 | 27 | yes | Ataxia |
| A01 | 27 | no | Ataxia |
| A02 | 58 | yes | Ataxia |
| A02 | 30 | yes | Ataxia |
| A03 | 34 | yes | Ataxia |
| A03 | 10 | no | Ataxia |
| A04 | 43 | yes | Ataxia |
| A04 | 23 | no | Ataxia |
| A05 | 14 | no | Ataxia |
| A05 | 14 | no | Ataxia |
| A06 | 23 | no | Ataxia |
| A06 | 9 | no | Ataxia |
| A07 | 12 | no | Ataxia |
| A07 | 17 | no | Ataxia |
| A08 | 36 | yes | Ataxia |
| A08 | 28 | yes | Ataxia |
| A09 | 14 | no | Ataxia |
| A09 | 10 | no | Ataxia |
| A10 | 12 | no | Ataxia |
| A10 | 20 | no | Ataxia |
| A11 | 13 | no | Ataxia |
| A11 | 29 | no | Ataxia |
| A12 | 35 | no | Ataxia |
| A12 | 28 | no | Ataxia |
| A13 | 41 | yes | Ataxia |
| A13 | 62 | no | Ataxia |
| A14 | 24 | yes | Ataxia |
| A14 | 14 | no | Ataxia |
| A15 | 62 | yes | Ataxia |
| A15 | 14 | no | Ataxia |
| A16 | 12 | yes | Ataxia |
| A16 | 12 | no | Ataxia |
| A17 | 17 | no | Ataxia |
| A17 | 34 | no | Ataxia |
| A18 | 13 | yes | Ataxia |
| A18 | 13 | no | Ataxia |
| A19 | 29 | no | Ataxia |
| A19 | 13 | no | Ataxia |
| A20 | 23 | no | Ataxia |
| A20 | 18 | no | Ataxia |
| A21 | 20 | no | Ataxia |
| A21 | 23 | no | Ataxia |
| A23 | 14 | no | Ataxia |
| A23 | 31 | no | Ataxia |
| A24 | 20 | no | Ataxia |
| A24 | 19 | no | Ataxia |
| A25 | 13 | no | Ataxia |
| A25 | 14 | no | Ataxia |
| A26 | 35 | no | Ataxia |
| A26 | 15 | no | Ataxia |
| A27 | 18 | no | Ataxia |
| A27 | 32 | no | Ataxia |
| C01 | 45 | yes | Control |
| C01 | 83 | yes | Control |
| C02 | 35 | yes | Control |
| C02 | 13 | no | Control |
| C03 | 53 | yes | Control |
| C03 | 46 | no | Control |
| C04 | 10 | no | Control |
| C04 | 18 | no | Control |
| C05 | 45 | yes | Control |
| C05 | 31 | no | Control |
| C06 | 58 | yes | Control |
| C06 | 72 | no | Control |
| C07 | 19 | no | Control |
| C07 | 13 | no | Control |
| C08 | 22 | no | Control |
| C08 | 20 | no | Control |
| C09 | 26 | no | Control |
| C09 | 23 | no | Control |
| C10 | 23 | yes | Control |
| C10 | 43 | yes | Control |
| C11 | 52 | yes | Control |
| C11 | 52 | yes | Control |
| C12 | 37 | yes | Control |
| C12 | 79 | yes | Control |
| C13 | 10 | no | Control |
| C13 | 35 | no | Control |
| C14 | 53 | yes | Control |
| C14 | 25 | no | Control |
| C15 | 40 | yes | Control |
| C15 | 29 | no | Control |
| C16 | 13 | yes | Control |
| C16 | 69 | no | Control |
| C17 | 36 | yes | Control |
| C17 | 22 | no | Control |
| C18 | 15 | no | Control |
| C18 | 16 | no | Control |
| C19 | 26 | yes | Control |
| C19 | 30 | no | Control |
| C20 | 28 | yes | Control |
| C20 | 13 | no | Control |
| C21 | 71 | yes | Control |
| C21 | 37 | no | Control |
| C22 | 44 | yes | Control |
| C22 | 41 | no | Control |
| C23 | 47 | yes | Control |
| C23 | 34 | yes | Control |
| C24 | 32 | no | Control |
| C24 | 32 | no | Control |
| C25 | 10 | no | Control |
| C25 | 16 | no | Control |
| C26 | 26 | yes | Control |
| C26 | 23 | no | Control |
| C27 | 97 | yes | Control |
| C27 | 29 | no | Control |
| C28 | 67 | yes | Control |
| C28 | 42 | no | Control |
